# Quantitative imaging feature pipeline: a web-based tool for utilizing, sharing, and building image-processing pipelines

**DOI:** 10.1117/1.JMI.7.4.042803

**Published:** 2020-03-14

**Authors:** Sarah A. Mattonen, Dev Gude, Sebastian Echegaray, Shaimaa Bakr, Daniel L. Rubin, Sandy Napel

**Affiliations:** a Stanford University, Department of Radiology, Stanford, California, United States; b The University of Western Ontario, Department of Medical Biophysics, London, Ontario, Canada; c The University of Western Ontario, Department of Oncology, London, Ontario, Canada; d Stanford University, Department of Electrical Engineering, Stanford, California, United States; e Stanford University, Department of Medicine, Stanford, California, United States; f Stanford University, Department of Biomedical Data Science, Stanford, California, United States

**Keywords:** medical image analysis, radiomics, machine learning, feature extraction, processing pipeline

## Abstract

Quantitative image features that can be computed from medical images are proving to be valuable biomarkers of underlying cancer biology that can be used for assessing treatment response and predicting clinical outcomes. However, validation and eventual clinical implementation of these tools is challenging due to the absence of shared software algorithms, architectures, and the tools required for computing, comparing, evaluating, and disseminating predictive models. Similarly, researchers need to have programming expertise in order to complete these tasks. The quantitative image feature pipeline (QIFP) is an open-source, web-based, graphical user interface (GUI) of configurable quantitative image-processing pipelines for both planar (two-dimensional) and volumetric (three-dimensional) medical images. This allows researchers and clinicians a GUI-driven approach to process and analyze images, without having to write any software code. The QIFP allows users to upload a repository of linked imaging, segmentation, and clinical data or access publicly available datasets (e.g., The Cancer Imaging Archive) through direct links. Researchers have access to a library of file conversion, segmentation, quantitative image feature extraction, and machine learning algorithms. An interface is also provided to allow users to upload their own algorithms in Docker containers. The QIFP gives researchers the tools and infrastructure for the assessment and development of new imaging biomarkers and the ability to use them for single and multicenter clinical and virtual clinical trials.

## 1 Introduction

The field of quantitative imaging is rapidly growing, especially in the area of radiomics and machine learning. Radiomics aims to extract quantitative image features from medical images to identify valuable biomarkers of underlying cancer biology.^1^^–^^4^ These features in combination with machine learning algorithms can be used for diagnosis and to predict clinical outcomes and/or treatment response.^5^^–^^7^ In addition, association of these imaging features with cancer genomics or other patient information may further describe the fundamental biology.^8^^–^^10^ Currently, quantitative image analysis tools are being developed for all disease sites and several imaging modalities to assess outcomes, diagnoses, and/or responses.^11^^–^^15^ However, current radiomics tools are lacking sufficient evaluation and validation, and there is a lack of translation of these tools into the clinical workflow. This is in part due to the lack of available shared software algorithms and architectures to fully compare and evaluate these quantitative imaging tools across institutions. Similarly, researchers must also have expertise in writing software code to perform many image analysis tasks, including radiomic feature extraction and machine learning. Currently, many open-source pipelines, including Slicer Radiomics,^16^ Orange,^17^ and KNIME,^18^ only process a single image at a time or only perform one quantitative imaging task, such as feature extraction or machine learning.

Therefore, what is critically needed is a user-friendly platform for sharing and assessing quantitative imaging algorithms. The quantitative imaging feature pipeline (QIFP) is an open-source, web-based platform that allows users access to a wide range of image processing and analysis tools without requiring writing code. Users are also able to upload their own algorithms in Docker containers, which allows the system to evolve and to support code that has been written in a variety of languages. This pipeline gives researchers the tools and infrastructure needed to assess and compare the value of combinations of quantitative image features. For example, researchers may want to create a pipeline that first performs segmentation of a region of interest, then performs feature extraction, and finally trains a machine learning classifier to predict an outcome of interest. The QIFP system allows users to complete these tasks in a single pipeline. It can also allow for the widespread development, assessment, and dissemination of new imaging biomarkers, including the opportunity for external validation of existing software pipelines. This system can also be used to facilitate incorporating quantitative imaging tools into single and multicenter clinical and virtual clinical trials specifically involving image processing, radiomics, and/or machine learning. The QIFP could be used as a central webserver where multiple institutions could upload de-identified imaging data and perform standardized image-processing pipelines.

## 2 Architecture

**Fig. 1:**
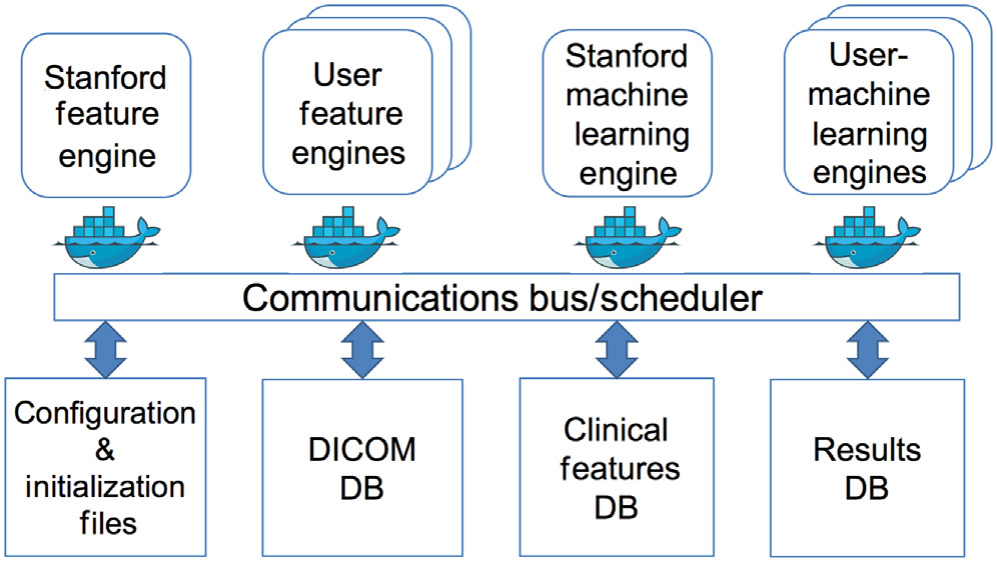
QIFP architecture. The top half of the figure represents a Docker image capable of given task, such as feature extraction or machine learning. The lower half shows the connections to run configuration options files and to various local databases, such as DICOM images/segmentations, clinical features, or workflow results.

Figure 1 shows the architecture of the QIFP, which uses the Common Workflow Language (CWL) execution model and the CWL standard for defining tools and workflows. Simple CWL (json) formatted definitions of tools or workflows can be imported into or exported from the QIFP system. The QIFP leverages Docker for ease of sharing algorithms that have been written in a variety of languages and on a variety of platforms.^19^ The entire QIFP is also available as a Docker version for installation on a local server to run within an institutional firewall. The server needs to have at least 4 cores and 64 GB of memory. A detailed user guide is available on the QIFP website^20^ under documentation, which provides details on how to perform this installation, including the required Docker-composed file.

The QIFP system, a web application written in java, runs under a Tomcat webserver. When running the QIFP through the webserver, there are no configuration or minimum bandwidth requirements; however, data upload and download speed will be determined by the user’s local network. The QIFP schedules and monitors tools to be executed by a workflow. Each of the blocks in the top half of Fig. 1 represents a Docker image capable of, e.g., feature extraction, image conversion, or machine learning. The system acquires the image, semantic, and clinical data from one of various sources [e.g., user’s computer, local database, the electronic Imaging Device (ePAD) system,^21^ and the Cancer Imaging Archive (TCIA)^22^]. The appropriate Docker images are then scheduled to run with the input images and clinical data or with the output of a previously scheduled Docker image as input. After each tool has completed, the system stores the output of the tool in the local database and schedules the next tool to be run as defined by the workflow. Once the workflow has completed, it sends an email to the user with a link to the results. The lower half of Fig. 1 shows connections to run configuration options files and various local databases.

## 3 Interface

**Fig. 2:**
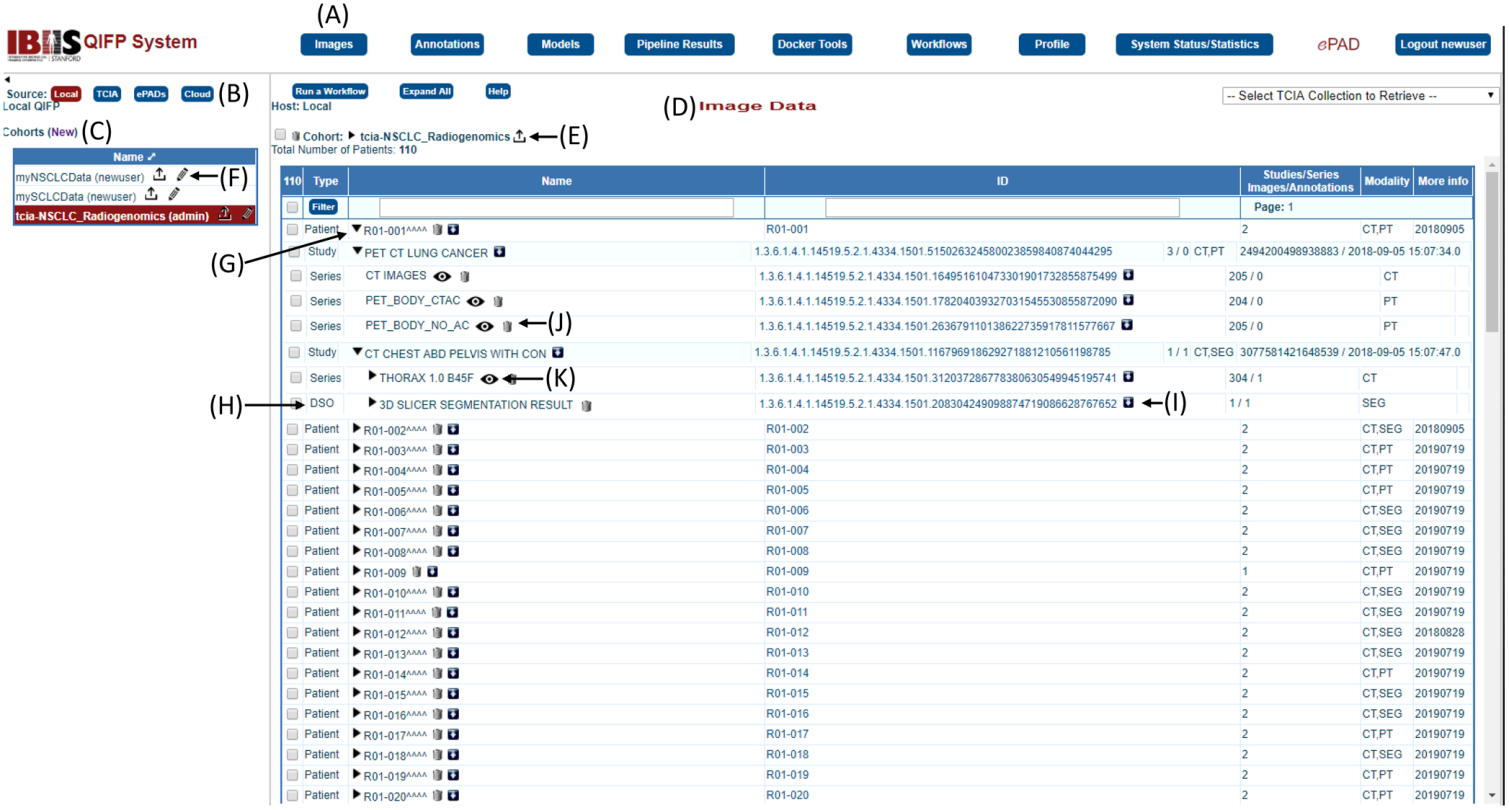
(A) QIFP interface with the “Images” menu displayed. (B) This example shows the data sources available, with the “Local” data source selected in red. (C) Three local cohorts are available (“myNSCLCData,” “mySCLCData,” and “TCIA NSCLC_Radiogenomics”) with the latter selected. (D) The list of patients in the TCIA NSCLC_Radiogenomics cohort is displayed in the “Image Data” section. (E) Clicking the arrow next to the cohort name allows user to upload images and/or segmentations. (F) Clicking the pencil will allow you to edit details regarding the cohort, including adding or removing other users to this cohort. (G) Clicking the triangle next to the patient name expands all available data for that patient, including studies, series, and annotations. (H) The annotation for this patient (3-D Slicer Segmentation Result) is a DICOM segmentation object. (I) Data can also be downloaded with the down arrow next to the ID or (J) deleted by clicking on the trash can next to the name. (K) Clicking the eye symbol next to an image series opens it in an ePAD image viewer within the window for viewing and potential annotation.

The QIFP is an open-source, web-based system publicly accessible at Ref. 20. After logging into the system with a distinct username and password, users will see the QIFP interface as shown in Fig. 2. Users can request an account on the main login page for the QIFP. Image cohorts are displayed on the left-hand panel and users can choose any of the top menu functions, as described below.

### 3.1 Images Menu

The QIFP maintains its own image repository and can connect to other image sources. Figure 2 shows the QIFP user interface when the “Images” menu (along the top row) and the “Local” source are selected, revealing a list of available cohorts in the left-hand panel and the available patient images/annotations in the right-hand panel. Other available sources include: The Cancer Imaging Archive (TCIA),^22^ an instance of the ePAD^21^ image annotation and storage system, and either the Google or Amazon S3 cloud services [Fig. 2(B)]. QIFP can also be configured to query data from any DICOM compliant local PACS. When QIFP is running on a local server, local cohorts can be created by transferring them from the other sources to the QIFP, or by manually uploading images, segmentations, and/or annotations by clicking on the upload button next to cohort name [Fig. 2(E)]. This will allow users to select a file type to upload and browse to files on their local computer. For example, users can upload a zip file of DICOM or Neuroimaging Informatics Technology Initiative (NIfTI) images and segmentations. Finally, owners of a local cohort can add other QIFP users to the cohort to allow them to access the data and workflows associated with it [Fig. 2(F)].

Once a cohort has been selected, the QIFP shows the list of DICOM images available in that cohort in the main right-hand panel and lists information on the patient, study, and series. A link to an ePAD image viewer (a freely available open-source DICOM viewer^21^) is also provided to quickly visualize an image series [Fig. 2(K)] and annotate images. Any new annotations (e.g., segmentation seed points) created in ePAD will then be available in the QIFP. Any annotations associated with a specific series are also displayed. Users can select individual series, studies, or patients to process by selecting the box next to each one. Otherwise, users can select the whole cohort by clicking the box next to the cohort name at the top of the screen, or a subset of patients by clicking on each patient and holding down the shift key to select all patients in between. Selected cohorts can then be processed by one of many processing tools and pipelines, described in Secs. 3.5 and 3.6, respectively.

### 3.2 Annotations Menu

The “Annotations” menu lists all the available image annotations or segmentations for a given cohort. Annotation files can be stored as annotation and image markup (AIM)^23^ files or DICOM segmentation objects (DSO). Users can also upload other segmentation file types, e.g., NIfTI file format as described above [Fig. 2(E)],^24^ and save them to the local cohort. Only users who are members of the local cohort have access to these segmentations.

### 3.3 Models Menu

Whenever a new predictive model is created through a machine learning workflow, the user has the option to save it and include it in future workflows. The “Models” menu contains a list of previously constructed predictive models that are available in the QIFP system.

### 3.4 Pipeline Results Menu

**Fig. 3:**
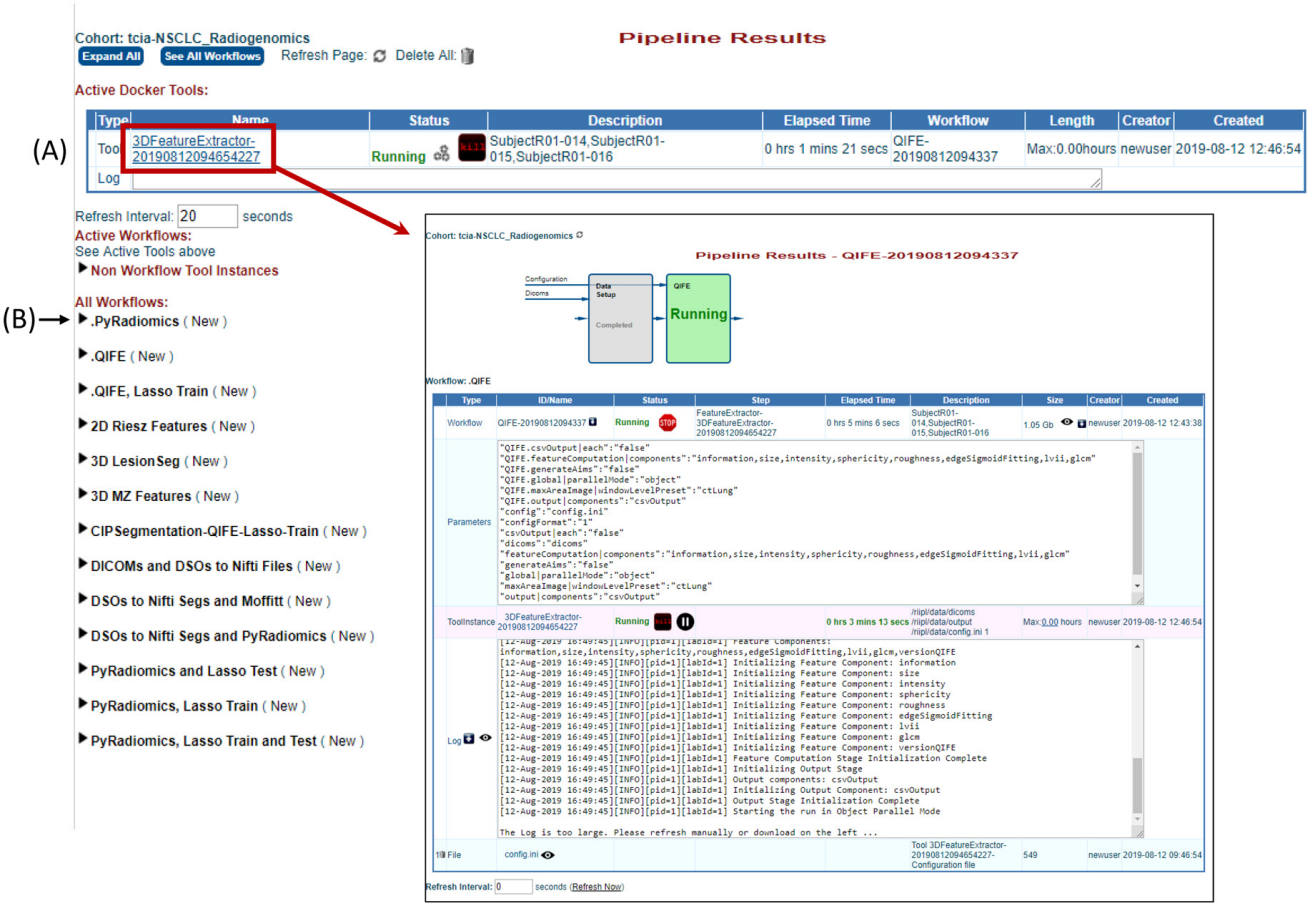
Example of the “Pipeline Results Menu” with a feature extraction workflow in progress. This menu shows all active and completed tools and workflows for the cohort selected prior to invoking this menu. (A) Currently active Docker tool (red box) and the results in progress, including status, elapsed time, parameters used, and access to a log file, pointed to by red arrow. Workflows shown below “Active Docker Tools” are organized by type; for example, clicking the arrow (B) will show all workflows using the PyRadiomics Tool completed or in progress. Clicking on the workflow name, as shown in the red box, will open a new webpage with additional details on that workflow.

Users can see results for past and progress for currently running workflows under the “Pipeline Results” menu. Figure 3 shows an example of a workflow in progress. The cohort name is listed on the top left corner of the page and actively running workflows are displayed under “Active Docker Tools.”

While a workflow is running, the Pipeline Results page displays relevant information, such as the elapsed time, the parameters used, log-file entries, and overall status of the workflow. All workflows have an ID name which is based on the tool name (e.g., QIFE), and the date and time that the workflow was started. Users receive an email once the workflow has completed. A log file is also available to provide information on the completed workflow, if it finds an error, and why it may have failed.

### 3.5 Docker Tools Menu

The “Docker Tools” menu provides access to all available Docker tools in the system, as well as a “Tool Help.” Currently, the QIFP system has a range of tools available for quantitative image analysis, including preprocessing, segmentation, feature extraction, and machine learning prediction models (Table 1). What follows provides a detailed explanation of the tools currently available on the QIFP.

**Table 1:** Docker tools currently available on the QIFP.

Tool type	Tool name
Feature extraction	PyRadiomics
	2-D JJVector feature extractor
	3-D feature extractor (Mu Zhou)
	3-D feature extractor (QIFE)
	Moffitt feature extractor
	SIFT feature extractor
Machine learning prediction engines	LASSO train prediction engine
	LASSO test prediction engine
	LASSO randomization prediction engine
Preprocessing tools	Analyze segmentation to NIfTI conversion
	DICOM-RT to DSO conversion
	NIfTI to DSO conversion
	DICOMs to NIfTI conversion
	DSO to Nifiti conversion
	DICOM validation tool
Segmentation	Lung segmentation
	Tumor segmentation
	2-D lesion segmentation
	3-D lesion segmentation
	CIP DICOM 3-D segmentation
	CIP NIfTI 3-D segmentation
Other	CoLiAGe feature map
	Delta features

#### 3.5.1 Preprocessing tools

There are many different formats for images, segmentations, and annotations and not all tools can process all formats. Therefore, QIFP contains tools that can be used for file conversion. For example, there are tools to convert between image types (e.g., DICOM, NIfTI) and segmentation types (e.g., DICOM-RT, NIfTI, and DSO). There is also a tool if a user wishes to validate DICOM files to ensure all required DICOM tags are present prior to processing.^25^

#### 3.5.2 Segmentation

Currently, there are several segmentation algorithms implemented as Docker tools available on the QIFP. There are two-dimensional (2-D) (2D LesionSeg) and three-dimensional (3-D) (3D LesionSeg) level set-based tumor segmentation tools, which take as input the image and a polygon or long axis line within the lesion.^26^^,^^27^ There is also a Chest Imaging Platform (CIP) Lesion Segmentation tool for DICOM or NIfTI files written by the Applied Chest Imaging Laboratory (Brigham and Women’s Hospital). This tool takes an input image and one or more seed points on the lesion and outputs a segmentation of the lesion of interest.^28^ AIM files are required to provide these inputs and specific example templates are provided on QIFP when running the workflow.

#### 3.5.3 Feature extraction

**Fig. 4:**
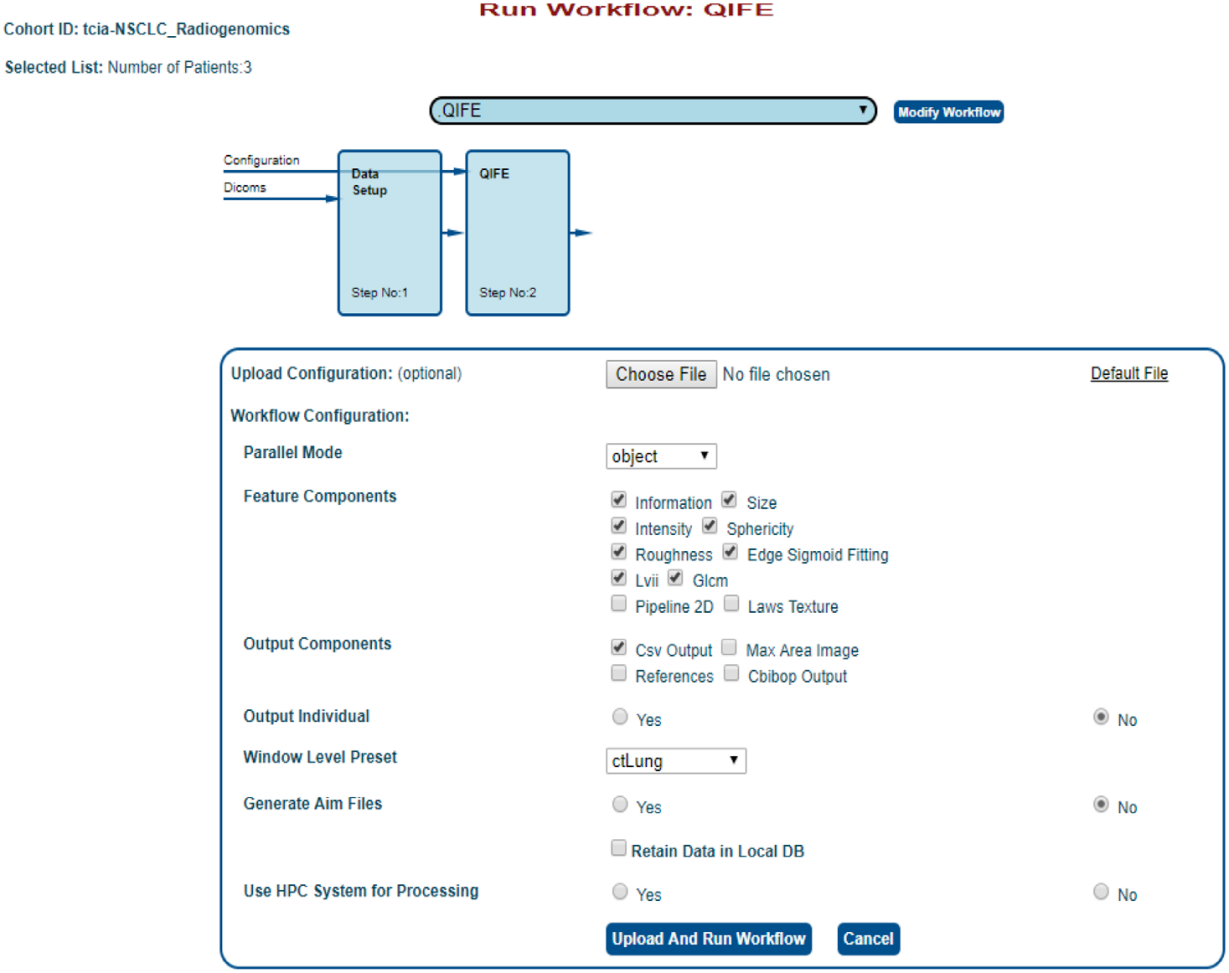
Example feature extraction workflow using the QIFE. Users can upload a configuration file or manually select configuration options in the interface shown.

**Fig. 5:**
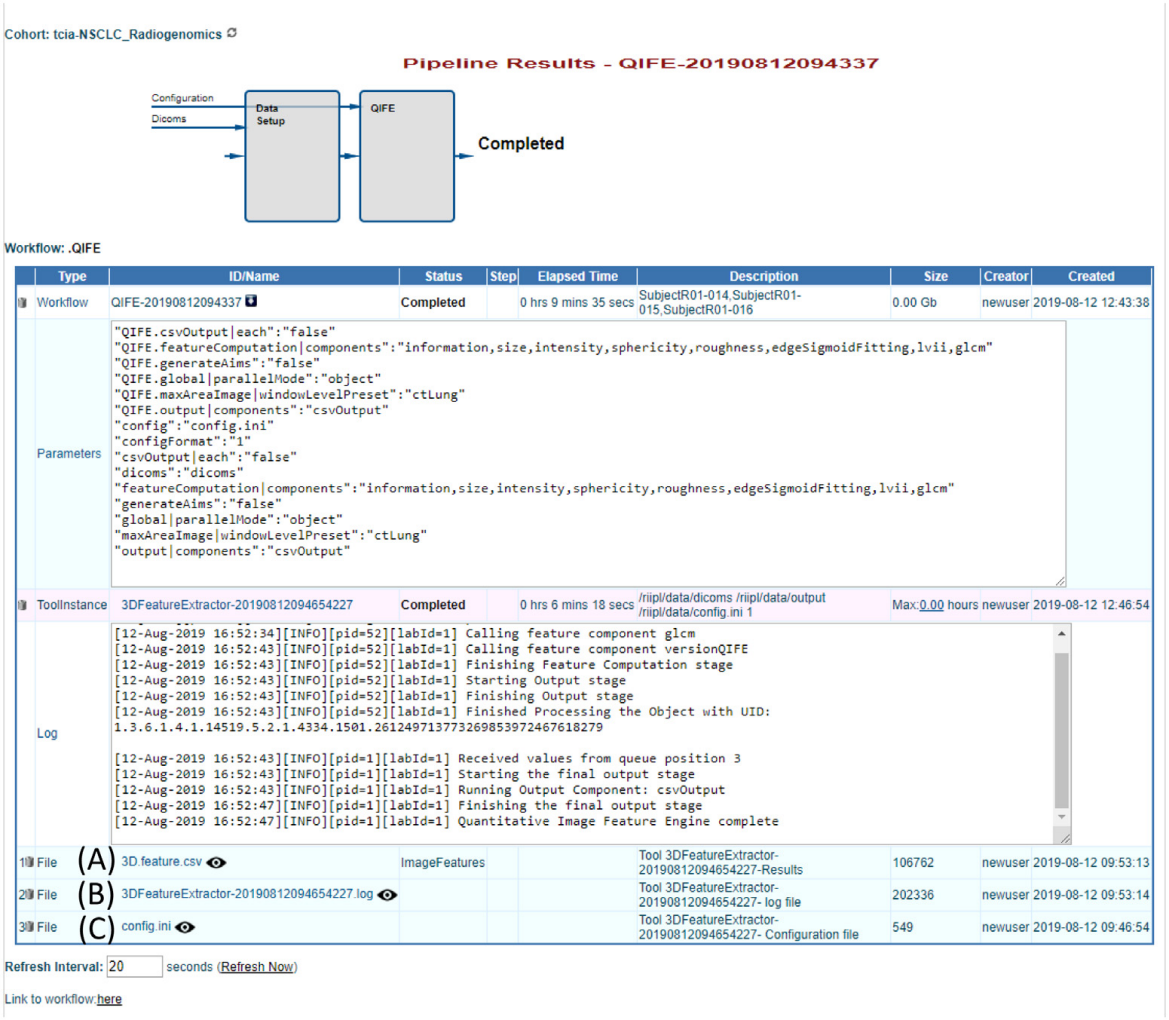
Example of a completed feature extraction workflow. The output components are displayed at the bottom. The files for this workflow include (A) the extracted features, (B) the log file describing the results of the workflow, and (C) the configuration file used to run the workflow. Clicking on any of the file names will allow the user to view and/or download them.

There are several different feature extraction modules that are currently available within the QIFP system. Stanford’s Quantitative Image Feature Extraction (QIFE) tool allows for the extraction of size, shape, intensity, texture, and law’s features.^29^ Figure 4 shows an example of how to configure a workflow containing this tool. Users can view, edit, and upload their own configuration file or manually select workflow options through the checkboxes provided. Figure 5 shows a completed workflow. All the files produced by the workflow are available for download through a link provided at the bottom of the results, including the log file, the resultant feature file in a comma-separated values (CSV) format, and the configuration file used for that run.

**Fig. 6:**
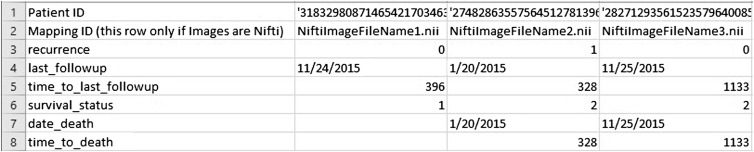
Example clinical file required in all LASSO training workflows.

The PyRadiomics tool is another feature extraction engine that has the option to extract higher order wavelet features along with the traditional features on the original images.^16^ Additional feature extraction tools include 2-D Riesz features^30^ and scale-invariant feature transform (SIFT) features.^31^ In general, each feature extraction module has its own set of user-configurable parameters to ensure the workflow is configured to best suit the required data type and analysis.

Different feature extraction modules may compute radiomic features differently, and for this reason may arrive at different values for what might appear to be the same feature.^32^ Common differences may be specifications for directional sampling of voxels for texture features, algorithms used for surface area calculations, and intensity discretization. We refer the user to the manuscripts describing QIFE^29^ and PyRadiomics^16^ for feature definitions. Also, since each Docker tool will contain a version of the tools from a specific point of time, the version code and Docker ID for each instance of the tool is recorded for each output workflow.

#### 3.5.4 Machine learning tools

Machine learning tools allow for the use of radiomic features with or without clinical features to predict an outcome or clinical parameter of choice (e.g., overall survival, specific gene mutation). The QIFP contains a least absolute shrinkage and selection operator (LASSO)^33^ tool, written using the open-source R software^34^ (Vienna, Austria). This tool can be configured for training and/or testing classification and regression models. To run a machine learning workflow, the user must also upload the corresponding clinical data and indicate the clinical parameter that they want to predict. An example file demonstrating how the clinical data should be organized is provided when the user sets up a workflow (Fig. 6). As with the feature extraction modules, each machine learning module has its own set of user-defined configuration parameters that can be used to customize the workflow. For example, the configuration file can specify the model type (binomial, Cox, etc.), the elastic-net mixing parameter alpha (LASSO to ridge), feature standardization, and number of folds for cross-validation. Future work is ongoing to add additional classifiers, hyperparameter tuning methods, and unsupervised machine learning techniques.

### 3.6 Workflows Menu

This menu allows the user to visualize all currently available workflows, which are also categorized according to their type, including feature extraction, segmentation, or prediction workflows (Table 2). User can also create a new workflow from modifications of any existing workflow. Workflows have already been created to run individual or combinations of Docker tools. For example, there is a workflow to run only the PyRadiomics feature extractor and another that will first run PyRadiomics followed by a machine learning engine. Workflows can be customized to include any of the tools listed in Table 1.

**Table 2:** Workflows currently available on the QIFP.

Workflow type	Workflow descriptions
QIFE 3-D/2-D features	All workflows that include the Stanford feature extraction code (QIFE)
PyRadiomics 3-D features	All workflows that include the PyRadiomics feature extraction code
Other 3-D features	All workflows that include feature extraction code other than QIFE and PyRadiomics
2-D features	All workflows that include feature extraction code for 2-D images
Prediction	All workflows that include the LASSO prediction tools
Image conversion	All workflows that include an image and/or segmentation conversion tool
Segmentation	All workflows that contain a segmentation tool
Other workflows	All workflows that do not fall into one of the above categories (e.g., semantic features)
All workflows	All workflows available on the QIFP

#### 3.6.1 Creating and customizing workflows

The simplest way to create a new workflow for existing Dockers tools on the QIFP is to run the existing “user configurable workflow.” This workflow allows users to manually select the Docker Tools to run, with the option to add/remove components. The configured workflow can then be saved with a new name for future use. Any existing workflow can also be customized by clicking on the “Modify Workflow” bottom beside the workflow name (an example is shown in Fig. 4).

### 3.7 Other Menus

The remaining buttons will provide access to user profile information and preferences (Profile) as well as usage statistics and event logs for completed QIFP actions (System Status/Statistics).

**Fig. 7:**
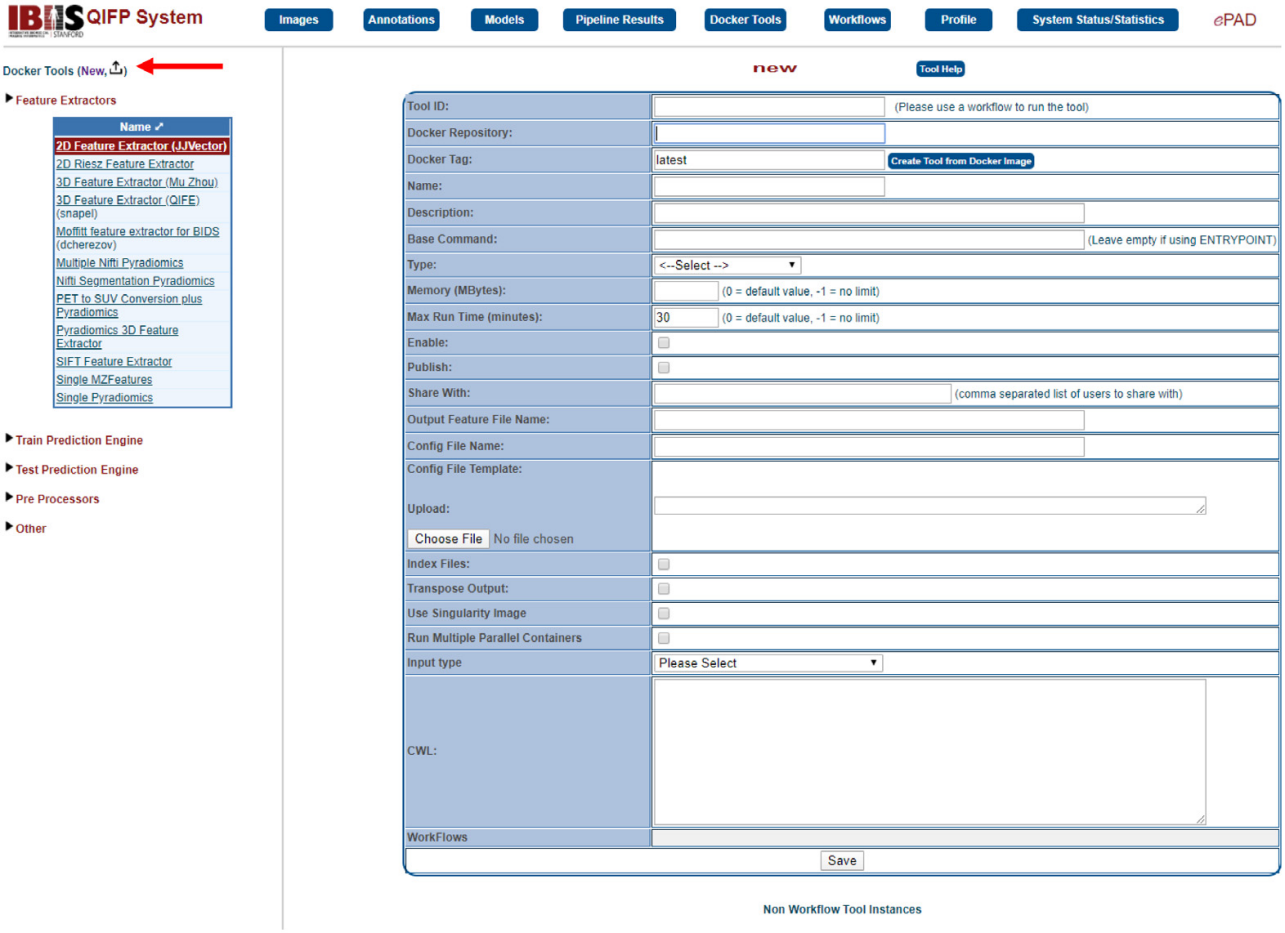
Example of how to add a new Docker Tool. After selecting the Docker Tool menu, click on the “New” option at the top of the left-hand (indicated by the arrow) to add the requirements for a user-supplied tool.

## 4 User-Supplied Tools

### 4.1 Creating and Uploading Tools

Users are able to upload their own tools to the QIFP by encapsulating them in a Docker container and storing them on DockerHub. For each Docker tool added to the QIFP, a Linux command should be indicated to describe the required inputs and outputs of the tool. For ease of incorporation into the QIFP system, tools can be created in two formats. The first being a tool that works on a single patient/series and a single segmentation or annotation. This type is simpler to implement, since the program does not need to figure out which segmentation refers to which series or to aggregate features/results for all patients. When using this type of tool on a whole cohort, the QIFP system will call the Docker image multiple times and run a separate Docker image for each case. The second option is to create a tool that processes multiple series/segmentations (i.e., an entire cohort). Each patient could have one or more series, and each series could have one or more segmentations/annotations. This type is more difficult to implement and requires using index files that contains file references and the feature results, if any, need to be aggregated into a single file. However, this type of tool is more efficient since only one Docker image is run for the entire data set. Figure 7 demonstrates how to upload a Docker tool and what information is required.

## 5 Example Workflow

This section provides a step-by-step example of how to run a workflow on the QIFP using publicly available data on the TCIA: the “NSCLC Radiogenomics” dataset^35^ processed by the PyRadiomics feature extraction module, followed by an LASSO predictive modeler. The goal of this example is to predict recurrence (a binary outcome) in this cohort of lung cancer patients. The clinical information for this example can be directly downloaded from the TCIA website.^36^ Users can request an account on the main login page for the QIFP.^37^

### 5.1 Selecting the Cohort and Workflow

**Fig. 8:**
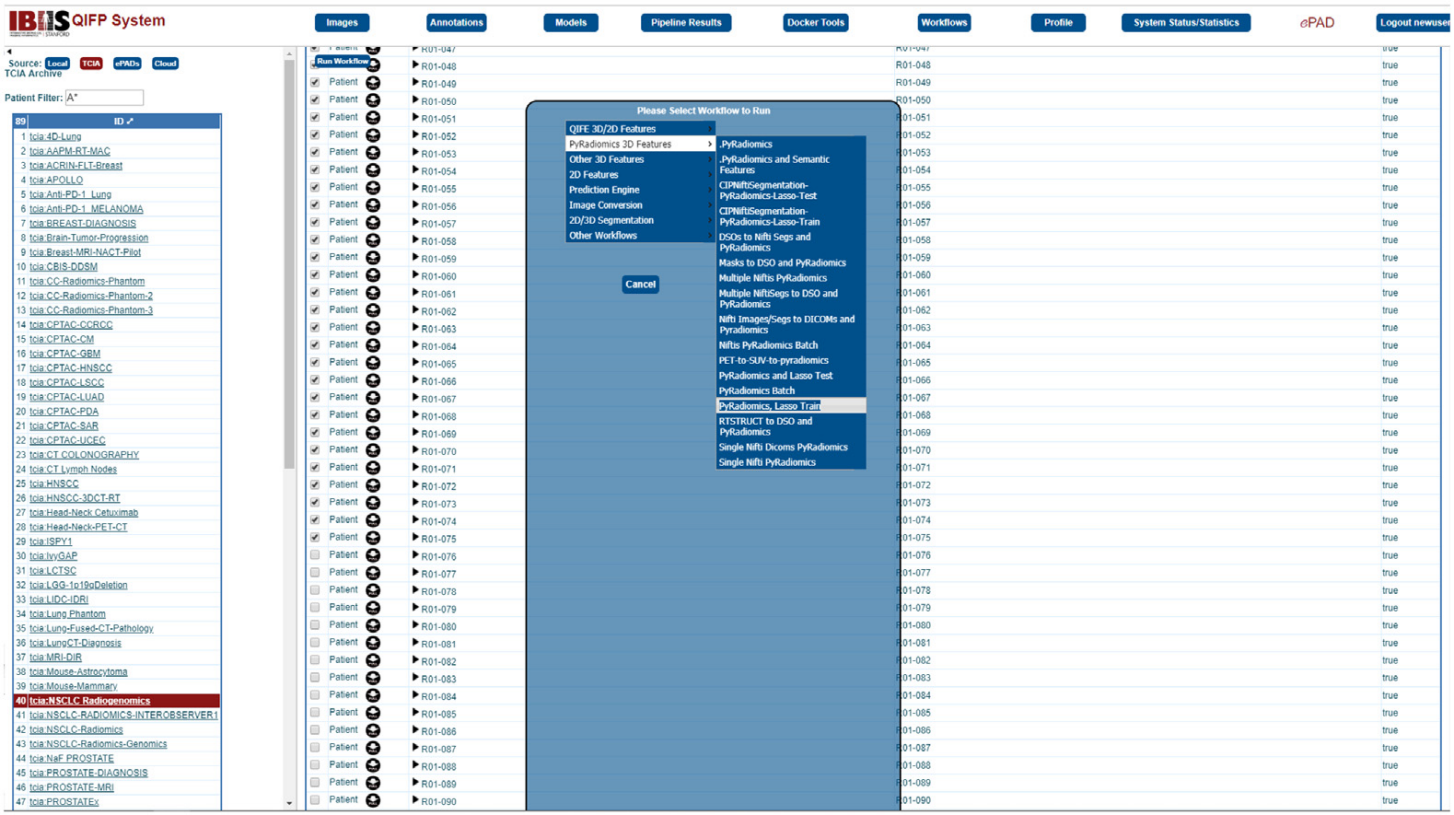
Example execution of a feature extraction and machine learning training workflow using the TCIA Radiogenomics cohort.

To run a workflow, the user must first select the cohort that they would like to analyze by clicking on “Images” menu at the top, choosing the “TCIA” source, and then clicking on the “tcia:NSCLC Radiogenomics” cohort. This example will process 75 patients (R01-001 through ROI-075). To select these, click on the checkbox next to R01-001 then scroll down to R01-075 then press and hold SHIFT while clicking on the checkbox next to R01-075. Next, click on “run workflow” at the top left-hand corner of the window and select the workflow of interest, in this example “PyRadiomics 3D Features → PyRadiomics, LASSO Train” workflow (Fig. 8). This workflow will first perform feature extraction using the PyRadiomics Docker tool and then perform LASSO training to build a predictive model.

### 5.2 Configuring and Running the Workflow

**Fig. 9:**
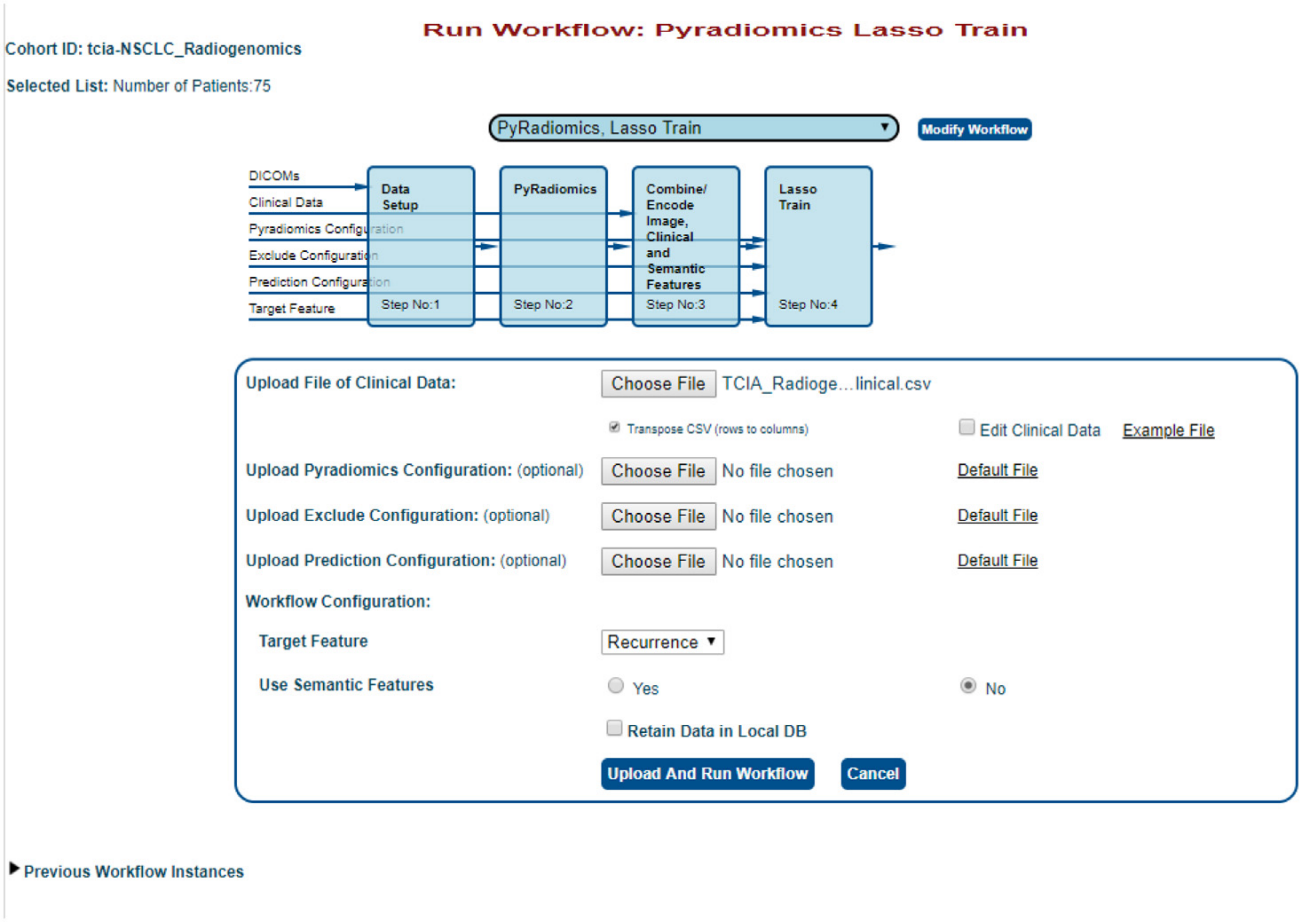
Input screen for the feature extraction and machine learning training workflow applied to the TCIA cohort. The required input requirements are shown to the left of the block diagram. Users have the ability to upload these required files and are provided with example files to illustrate formatting and default files used.

Figure 9 shows what information must be provided to successfully run the workflow. Specific text is shown to the left of the block diagram with arrows indicating which of these inputs are required for each specific tool. This includes uploading a file of clinical data, which includes the outcome of interest and a link to the image patient ID. For this example, extract columns A (case ID) and AE (recurrence) from the TCIA clinical data file. All rows for cases beginning with “AMC” can be deleted, so that only “R01” cases remain. The “Case ID” header must also be renamed to “Patient ID,” then these two columns can be saved as a new CSV file. This will be the clinical data file uploaded to run this workflow. In this case, the CSV file must be transposed, and the target feature “recurrence” can be selected from the drop-down menu provided. Configuration files for the feature extraction component and prediction engines can also be uploaded here; however, this example will use default configuration options. Users are also provided with output and processing options for each workflow. Selecting “retain data in local DB” will save the data in a local cohort named “tcia-NSCLC_Radiogenomics” and this will avoid having to redownload the images from TCIA for future processing. After all the selections have been made, clicking on “Upload and Run Workflow” will start the workflow. The status of the workflow can be tracked under the “Pipeline Results” menu. An email will be sent to the user once the workflow has completed with a direct link to the results of the workflow.

### 5.3 Saving the Prediction Model

**Fig. 10:**
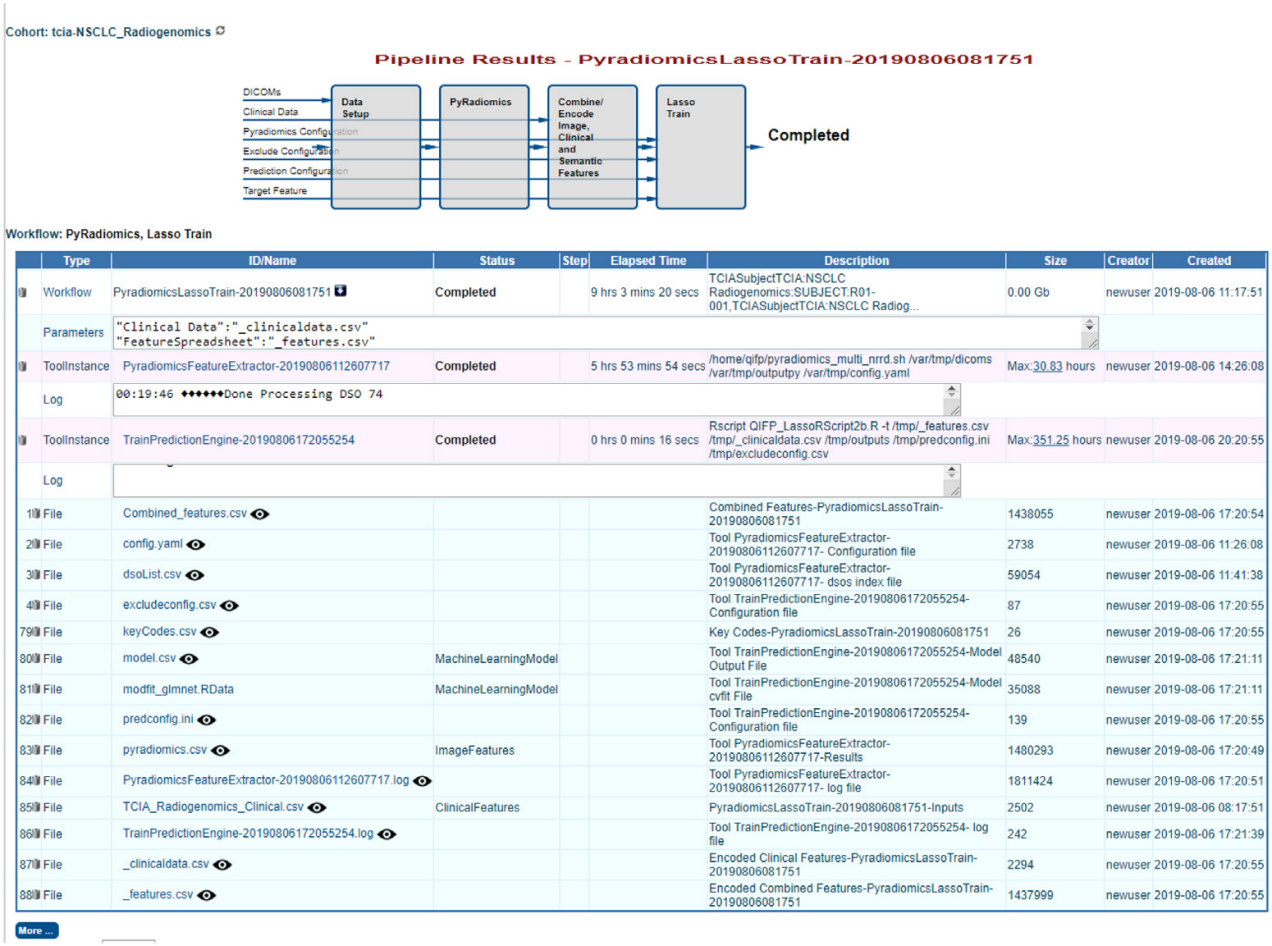
Output screen for the feature extraction and machine learning training workflow applied to the TCIA cohort (Fig. 9).

**Fig. 11:**
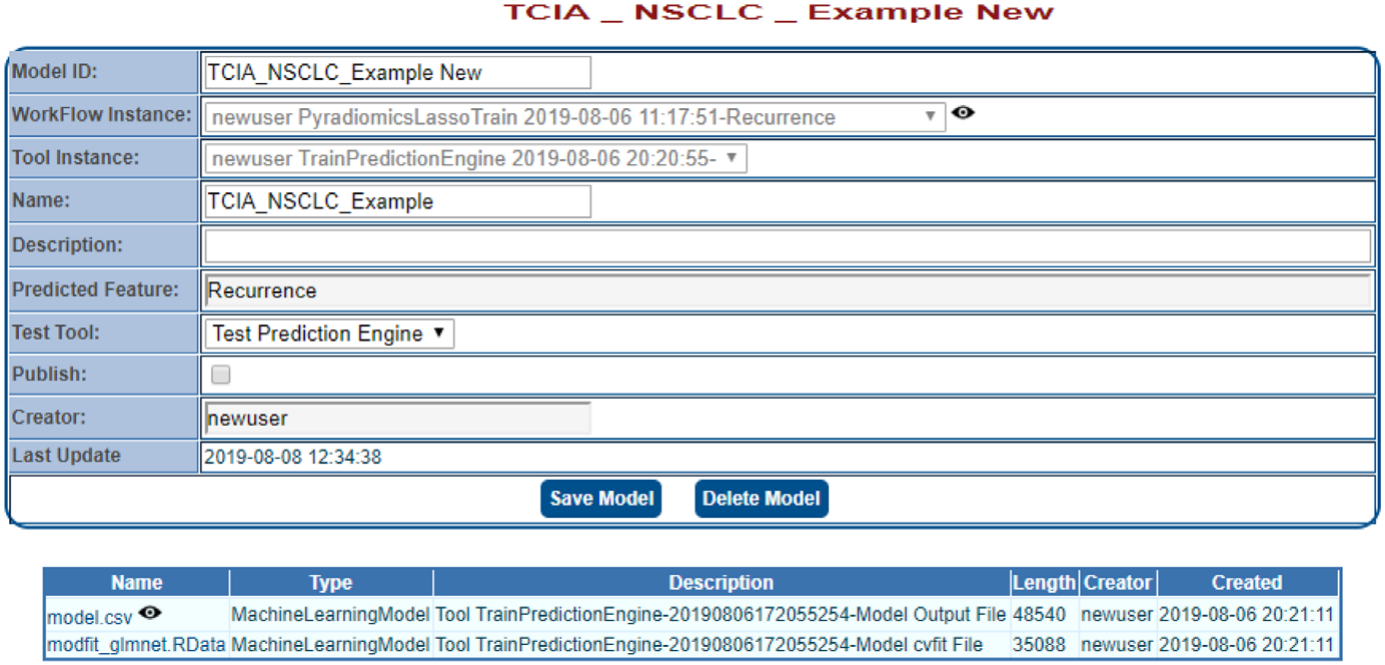
Output screen allowing the saving of the model produced by the workflow applied to the TCIA cohort.

The resultant model parameters can be found in the model.csv file provide as output from the training workflow (Fig. 10). In this example, LASSO selected six features for the final model. Figure 10 shows the output from the workflow, including the configuration files, feature extraction, and model results. To save the model and test it on a new cohort, users must go to the “Models” menu and click on “New” on the top of the left-hand panel. Users can name the model and select the appropriate workflow instance and tool instance from the training workflow that was just completed. Once a model is saved, it will appear under the “Models” menu on the left-hand side (Fig. 11).

### 5.4 Testing the Prediction Model

**Fig. 12:**
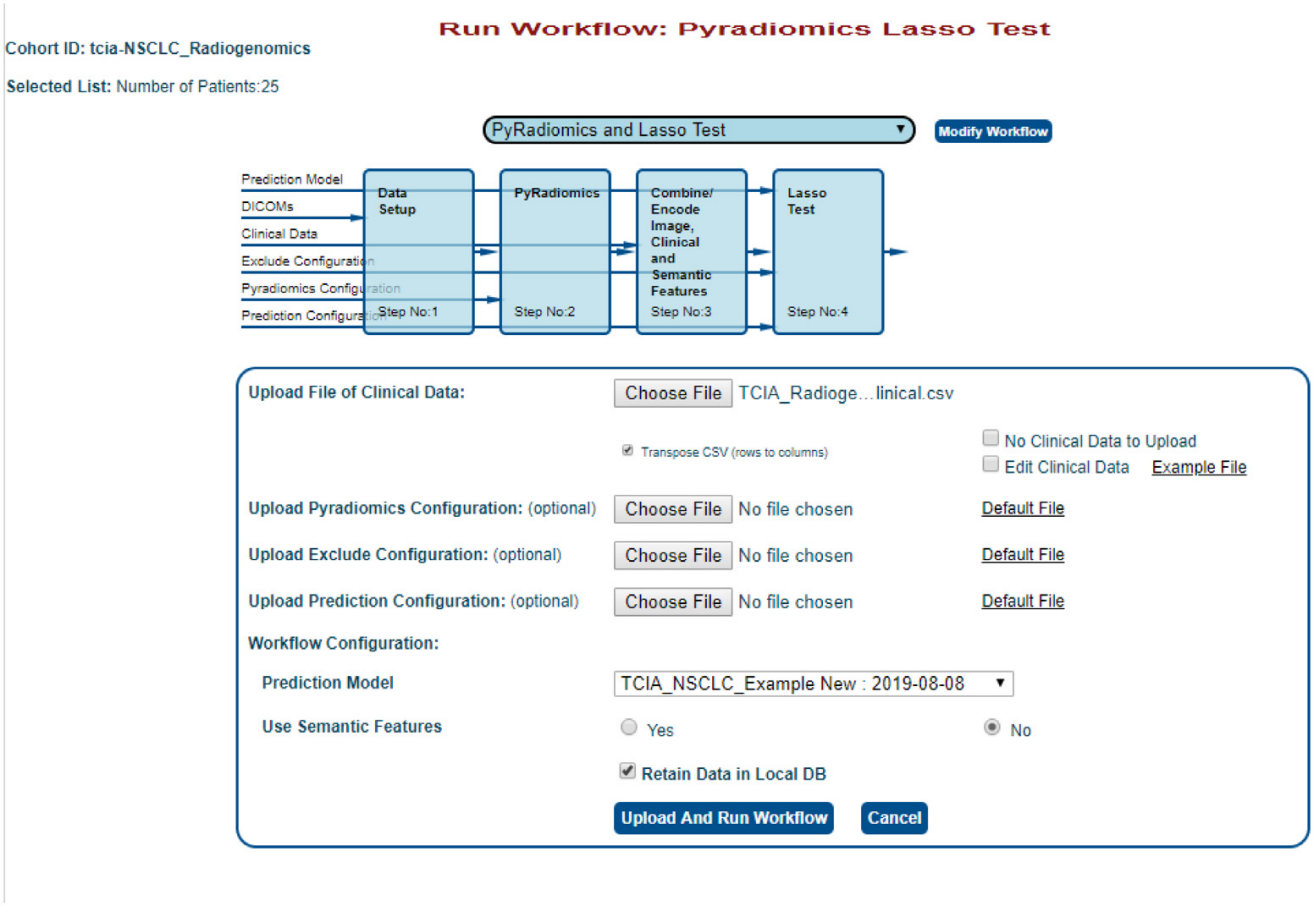
Input screen for the feature extraction and machine learning testing workflow applied to the TCIA cohort.

**Fig. 13:**
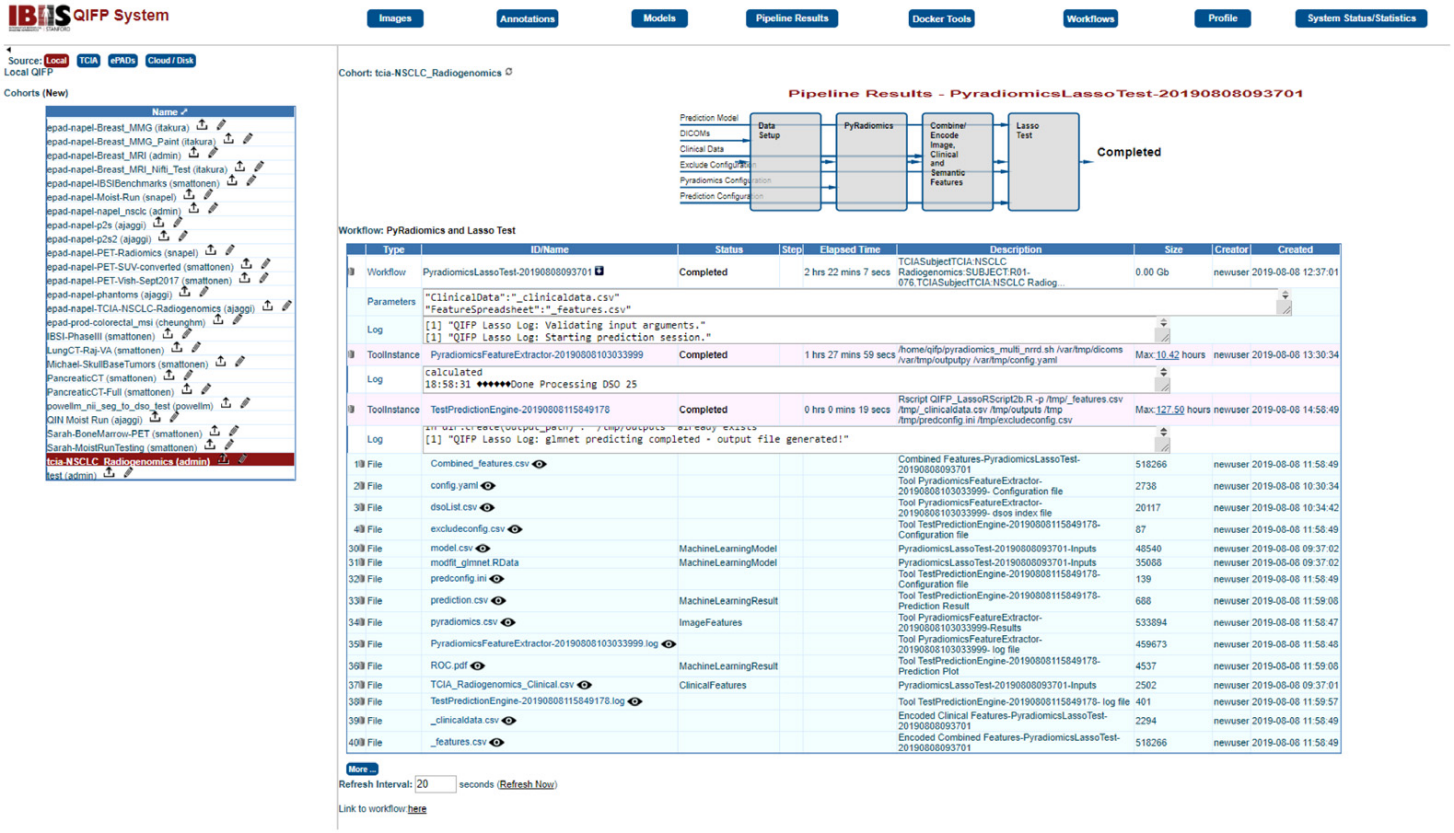
Output screen for the feature extraction and machine learning testing workflow applied to the TCIA cohort (Fig. 12).

**Fig. 14:**
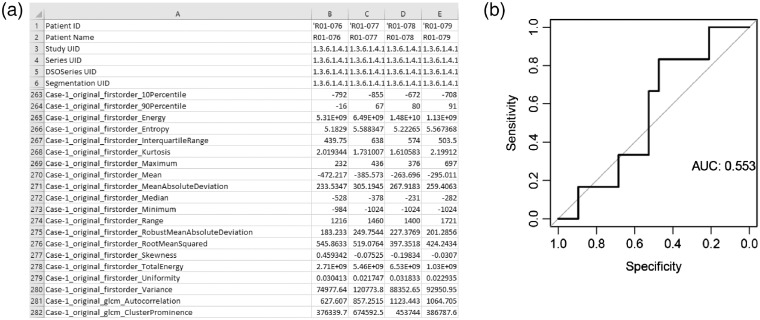
Resulting features and area under the ROC curve produced by the feature extraction and machine learning testing workflow applied to the TCIA cohort (Fig. 13). (a) The output pyradiomics.csv file displays all features in the rows and all images in the columns. The first six rows identify the annotation, rows 7 to 262 contain metadata and were removed from the figure. Quantitative features start at row 263 and a subset of 20 of the 900 features is shown. (b) The area under the ROC curve is 0.55. Note that this example is for illustrative purposes only, the cohorts have not been preselected, standardized, or balanced for the outcome of interest, and the performance of the classifier has not been optimized for this dataset, including assessing performance with time-to-event analysis.

To test this model on a new cohort of patients, patients are selected as described in Sec. 5.1. For this example, testing will be done on 25 different patients from the same TCIA NSCLC radiogenomics cohort (patients R01-076 through R01-100) by running the workflow “PyRadiomics 3D Features → PyRadiomics and Lasso Test.” The same clinical data file can be uploaded that was used for testing, since it includes all patients in the TCIA dataset (Fig. 12). Once again, the default feature extraction and prediction configurations will be used. The model that was saved in Sec. 5.3 above can be selected from the drop-down menu and then the workflow can be started. Once the workflow has completed, the output files will be displayed (Fig. 13), including a list of the resulting model’s features and their coefficients, and an area under the receiver operating characteristic (ROC) curve describing performance (Fig. 14).

## 6 Limitations and Future Work

Although the QIFP is equipped with several preprocessing and feature extraction tools, there is a limited number of machine learning tools available. Future work will include the addition of new feature selection methods and classifiers, including unsupervised machine learning techniques, as well as methods for hyperparameter tuning. Another limitation is that there are currently no cross-validation modules, including random sampling or bootstrapping; however, this is an area of ongoing work. There are also no deep learning tools available on the QIFP; however, since Docker easily allows for sharing algorithms, it would be relatively easy to dockize a pretrained neural net. Finally, the QIFP does not have any built-in data visualization or harmonization tools, important in quantitative imaging and, therefore, another area of future work.

## 7 Conclusions

The QIFP is an open-source, web-based platform that allows users to access, share, and build configurable quantitative image processing pipelines for both planar and volumetric medical images. The QIFP gives researchers the tools and infrastructure for the assessment and evaluation of new imaging biomarkers in single and multicenter clinical and virtual clinical trials. This includes performing all aspects of quantitative imaging, from segmentation to feature extraction and machine learning. The QIFP currently has 68 registered users across 18 institutions and companies in the United States, Canada, and Europe. Any researcher can request an account on the QIFP system using the link provided on the QIFP login page. A detailed user guide is also available on the QIFP website.^38^
